# Modern software and physical education: can online training enhance gym training?

**DOI:** 10.1186/s12909-024-05345-x

**Published:** 2024-04-18

**Authors:** Linna Ge, Menglu Li, ChangFeng Ning

**Affiliations:** Institute of Physical Education, Yancheng Institute of Technology, Yancheng, Jiangsu China

**Keywords:** Motivation, Physical activity, Physical education, Sprint, Squat jump, Technology, University students

## Abstract

**Background:**

This study discusses the effectiveness of a 12-week intervention aimed at improving squat jump and sprint performance among second-year sports students.

**Methods:**

The students were randomly divided into experimental (*n* = 89) and control (*n* = 92) groups. In addition to gym training, students of the experimental group also underwent online PE training. The students’ performance in Squat Jumps, 30 m sprint, and Progressive Aerobic Cardiovascular Endurance Run (PACER), as well as their situational motivation, were assessed before and after the intervention. Furthermore, the students assessed their physical activity weekly using self-reports.

**Results:**

The implementation of online training has positively impacted intrinsic and identified motivation, as well as external regulation; however, it was less effective in reducing amotivation compared to traditional gym-based training.

**Conclusions:**

The findings of the study contribute to the data synthesis on the expediency of using modern software in physical education.

## Introduction

Students, born after 2000, often referred to as “digital natives” [1], live in an era of new technologies that have revolutionized educational approaches and life itself. The debate about the effectiveness of using technological means to improve physical education (PE) started in the 1970s [2]. The quarantine restrictions of the COVID-19 pandemic have catalyzed the utilization of new technologies across various domains, including education and physical activity [3, 4]. Teachers and coaches have been compelled to swiftly adapt to the use of remote technologies and online resources [4]. For example, pedometers and accelerometers allow teachers to track the number of steps made by students. Heart rate monitoring devices track the intensity of student participation in a particular activity. Mobile apps provide a variety of educational tools and resources for presenting fitness information, creating assignments, and assessing physical activity (PA). Video materials shown on a large screen or online can support the teacher’s instructions or even replace them if necessary. In addition, modern students have the opportunity to improve their PA, cognitive functions, and learning outcomes via mobile exergames. This is a type of activity where a student is required to perform kinesthetic movements to play a video game [5].

At the same time, many researchers [3, 4, 6–10] emphasize that nowadays there is a decrease in physical activity (PA) among university students. They believe that this will inevitably lead to the deterioration of students’ physical fitness and general well-being [6]. The decreasing PA is mainly associated with an increased study load [6]. Another crucial factor that led to PA reduction among students was the COVID-19 quarantine restrictions, during which PA resources were limited [11–13].

On the one hand, there are plenty of tools and opportunities for PE classes and extracurricular activities that are designed to improve the PA of students. On the other hand, there is an aggravation of the decline in PA and deterioration of physical conditions. Such a contradiction suggests the need to revise existing approaches and search for more optimal strategies to implement technology-based training practices in higher education. The construct of perceived achievement is one of the most popular factors considered in PE [14]. Based on empirical research, scientists Bortoli et al. [15], Cid et al. [16], Huéscar Hernández et al. [17], and Vasconcellos et al. [18] concluded that higher levels of motivation determine higher academic performance in PE. Measuring students’ perceptions of the motivational climate during classes can provide teachers with the necessary tools to create and adapt PE lessons. This way, students can become increasingly engaged in educational activities and gain experience in achieving better results [14]. However, the lack of psychometric testing can lead to false achievements in sports. For that reason, it is necessary to explore PE not only in terms of achievements but also in terms of exercise motivation.

## Literature review

In studies related to physical activity (PA), researchers often refer to the Self-Determination Theory (SDT) [18]. According to SDT [19], motivation comprises four components: intrinsic motivation, identified regulation, external regulation, and amotivation. Intrinsic motivation is rooted in internal desire and satisfaction derived from the activity itself, identified regulation pertains to the conscious acceptance of the importance of an action, external regulation is determined by external incentives or punishments, whereas amotivation indicates a lack of motivation or desire to act [19].

Previous studies [20, 21] suggest that online training can support and enhance intrinsic motivation. Providing opportunities for choice in selecting workouts and interaction within the online environment fosters intrinsic motivation toward physical activity [20]. Research [22] also indicates that identified regulation can be strengthened through conscious awareness of the importance of the set task, which can be reinforced through online communication and training. External regulation, typically, can be enhanced by creating favorable conditions and rewards [20]. Some users may exhibit amotivation, particularly if there is a lack of adaptation to the online format. The utilization of new technologies, interactive elements, and program personalization can influence changes in users’ motivational orientations and contribute to improving physical activity [11, 23–28].

Fernández-Espínola et al. [28] in their systematic review considered the effects of interventions designed to increase PA of students. The interventions were also based on self-determination theory and achievement goals theory. Seven of the eleven studies reviewed by Fernández-Espínola et al. [28] reported increased student PA upon interventions. The authors explain such effect of interventions through well-chosen learning strategies, family involvement, and the use of motivational videos that promote PA.

Rodrigues et al. [14] studied motivational climates in PE oriented by teachers, peers, as well as behavioral regulations. The authors documented significant correlations between motivational climates and behavioral regulations were confirmed [14, 29]. Likewise, Behzadnia et al. [30] described the experience of an intervention that aimed to support students’ autonomous motivation in PE. An additional goal was to determine whether autonomous motivation would contribute to students’ motor skills in badminton. The results of Behzadnia et al. [30] confirmed the effect of the intervention. Specifically, the experimental group showed better badminton performance and reported significantly higher levels of autonomous motivation.

Lu et al. [25] and Gu et al. [24] employed the goal-setting theory. This theory investigates the impact of setting specific goals on motivation and achievement. Lu et al. [25] examined the effects of a 12-week resistance training program and goal-setting on adolescents, identifying goal-setting as a motivational tool. In contrast, Gu et al. [24] utilized a pedometer-based intervention strategy combined with goal-setting to study motivation in physical education and physical activity among schoolchildren.

In their study, Mokmin and Jamiat [27] designed and tested an app that consists of five virtual fitness trainers. These trainers provide exercises of different complexity depending on a student’s baseline level of physical fitness. Upon completion of the study, the authors found an increase in motivation among students. The students themselves reported being more eager to learn and more satisfied with the learning process [27]. In addition, the students agreed that the app improved their fitness and motivated them to perform fitness activities [27].

As we can see, there are many scientific publications devoted to the topic of using technological innovations to improve students’ PA. Only a few studies [25, 31] focused on a comprehensive approach that would consider both physical training and behavioral changes in the context of PA. However, it is important to note that these studies concerned school children, not university students. The current study aims to narrow this gap and optimize the physical training approach for university students by incorporating an online PE course. Here are the main research questions:

### RQ1

Will the training positively affect participants’ PA?

### RQ2

Will online training increase the students’ exercise motivation?

### RQ3

What is the relationship between the students’ PA, demographics, and academic performance?

## Methods and materials

### Study design and procedure

**Fig. 1:**
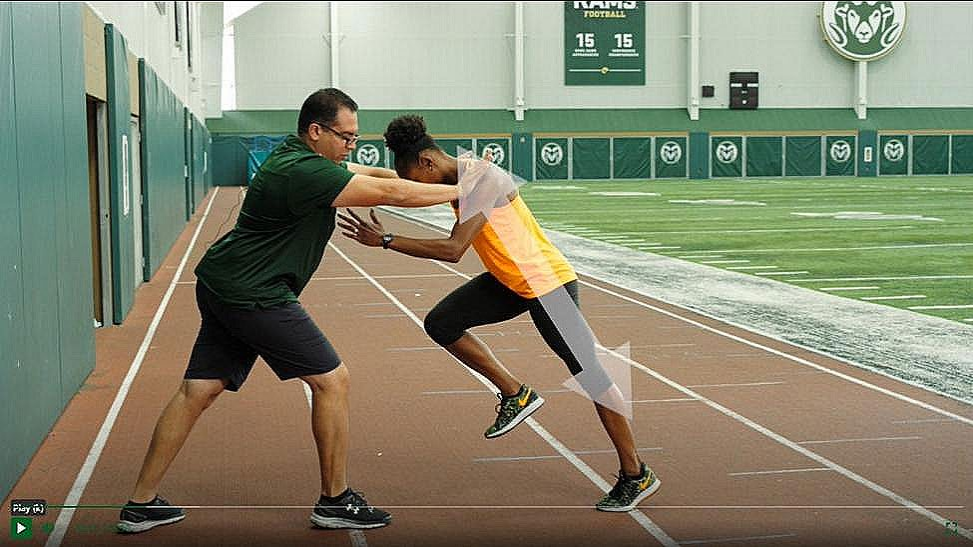
Screenshot of the video lesson used in the course

The study uses a pre-test and post-test quasi-experimental design. The study was conducted with the help of sports science students from two Chinese universities who took the online training course “Sprinting Smarter, Sprinting Faster”. The course included 34 video lessons (Fig. 1), a study guide, bonus materials, examples of sprint training with strength standards, a stride length calculator, and speed exercises. The total duration of the video lessons was 2 h and 6 min.

**Fig. 2:**
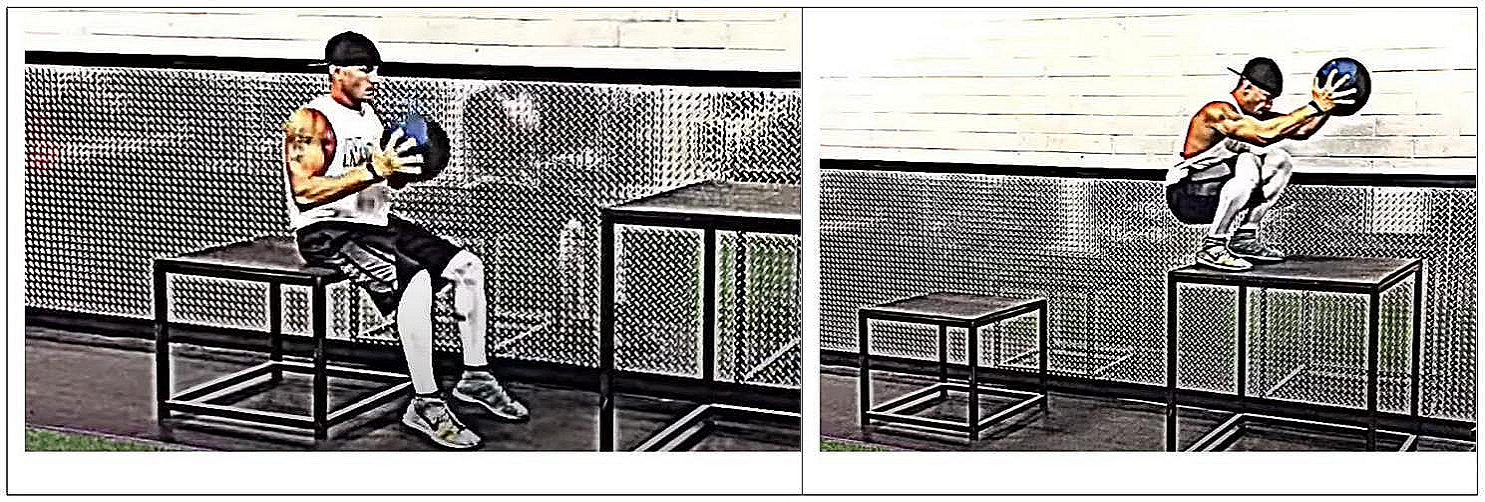
Exercise for practicing the starting strength in the gym Source: “Athlete Factory” YouTube channel [32]

The results of the experimental group who took the online course were compared with the results of the control group that studied according to the standard curriculum. The gym training of both groups involved practicing sprinting at the optimal speed as well as mastering different types of jumps. Thus, the students practiced:

• Starting strength: jumping on a stand without using the arms (Fig. 2) [32];
• Accelerative strength: strength-focused exercises with an average velocity of 5 to 7 m/s;
• Reactive strength: a depth jump over a hurdle. The hurdle reduces the ground contact time and increases the power output of the jump.

The participants had to attend 3 classes a week. Additionally, they had an opportunity to choose the days and hours during which they would attend the classes. The total duration of the intervention was 12 weeks (October-December 2022). To be included in the study, a respondent had to: (1) be a second-year student; (2) obtain a certificate for completing the online course; (3) give written consent to participate in the study; (4) have no physical or mental diseases in the medical record.

### Sample

The study involved 181 sports science students from two Chinese universities. The experimental group consisted of 89 students, the control group − 92 students. Parameters such as height, weight, and age of the respondents were obtained from their medical records. Demographic data of the participants are presented in Table 1.

**Table 1:** Students’ demographics

	Sex	Height (cm)	Weight (kg)	Age
Experimental group	Male	173.86 ± 5.45	66.12 ± 8.41	19.06 ± 1.00
	Female	161.52 ± 5.66	55.39 ± 4.94	19.32 ± 0.81
Control group	Male	174.08 ± 6.03	67.42 ± 10.12	19.54 ± 0.77
	Female	161.26 ± 5.73	54.10 ± 8.19	19.21 ± 0.84

In the experimental group, the average age of boys was 19.06 ± 1.00 years, with a height of 173.86 ± 5.45 cm and a weight of 66.12 ± 8.41 kg; for girls: the age was 19.32 ± 0.81 years, with a height of 161.52 ± 5.66 cm and a weight of 55.39 ± 4.94 kg. In the control group, the average age of boys was 19.54 ± 0.77 years, with a height of 174.08 ± 6.03 cm and a weight of 67.42 ± 10.12 kg; for girls: the age was 19.21 ± 0.84 years, with a height of 161.26 ± 5.73 cm and a weight of 54.10 ± 8.19 kg. The sample was homogeneous in terms of race and nationality; all participants were Chinese.

### Measurements

Physical Fitness Test consisted of Squat Jumps (SJ), a 30 m sprint, and the Progressive Aerobic Cardiovascular Endurance Run (PACER) test. Testing was conducted twice– at the baseline stage of the study (pre-test) and after the intervention (post-test). Prior to the testing, the participants performed a 15-minute warm-up.

The Optojump platform was used to determine the jump height. This platform has intraclass correlation coefficients for validity of 0.997, test–retest reliability of 0.982–0.989, and low random errors (2.81 cm) [33]. Only the best jump attempt out of three was considered during the jump height analysis. The sprint time was recorded using an electronic chronometer (Omega system), the accuracy of which is 0.001 [34].

The PACER test is performed before students reach the state of fatigue, which allows them to measure their peak aerobic power. Students run back and forth between two lines for as long as possible while staying 20 m apart from each other. The student moves from one point to another, the rhythm creates the sound signal. The test ends when the student reaches fatigue or fails to cross the line in time [33]. The speed achieved by the student at the final stage of the test is considered the final speed reached [33]. The reliability of the PACER test is very high (*r* = 0.97) [34].

Physical activity and exercise motivation were assessed using the self-administered short form of the International Physical Activity Questionnaire (IPAQ-7) [35]. The IPAQ-7 consists of 7 items that assess at least 10 min of PA (vigorous activity, moderate activity, and walking) in four different areas over the final three training days. These PA areas include transportation, occupation, house/lawn, and leisure [35]. Total weekly PA time is the total time the students spent performing vigorous activity, moderate activity, and walking [35]. The IPAQ-7 questionnaire was completed by the students weekly. We have informed the experimental group students that the questionnaire should be completed without taking into account their PA during training. However, the students considered the PA from all their sports science classes.

Exercise motivation was determined using the Situational Motivation Scale (SIMS), proposed by Guay et al. [36]. The SIMS contained 16 items that had to be rated on a scale from 1 to 7. Items 1, 5, 9, and 13 corresponded to the Intrinsic motivation subscale (Cronbach’s alpha 0.86). Items 2, 6, 10, and 14 corresponded to the Identified regulation subscale (Cronbach’s alpha 0.82). Items 3.7, 11, and 15 - External regulation (Cronbach’s alpha 0.91); items 4, 8, 12, and 16– Amotivation (Cronbach’s alpha 0.88).

### Statistical analysis

Pearson product-moment correlation analysis was used to identify the relationship between variables (sex, height, weight, PA, results in jumping and running).

The population was analyzed using odds ratios, utilizing data on physical activity in both experimental and control groups before and after intervention. Odds ratios for each level of PA “before” and “after” were considered. Correlation coefficients were used to determine associations between pre- and post-motivation levels for different types of motivation. The Breslow-Day test was utilized to check for heterogeneity of correlations. Pre-intervention motivation levels were employed as the independent variable. This allowed determining whether there were statistically significant differences in correlation relationships between groups.

### Ethical issues

The study obtained approvals from the ethics committees of the universities where it was conducted (Protocol No. LT 12,271,472 and TR 7,435,691). Participants were provided with all necessary information regarding the nature and conduct of the research. All participants were adults and provided written consent to participate. No personal information was processed or retained.

## Results

### The effectiveness of the intervention regarding PA

For Vigorous PA: Control group Pre = 93.76, Post = 95.14, Experimental group Pre = 91.16, Post = 116.77 (Table 2). The odds ratio for Vigorous PA: 1.257. The probability of achieving a high level of physical activity after the intervention in the experimental group was 26% higher than in the control group. However, this value does not reach statistical significance (as it is not large and includes 1).

**Table 2:** Descriptive statistics for pre- and post-testing on PA in the experimental and control groups

		Pre-test		Post-test	
PA	Group	Mean	SD	Mean	SD
Vigorous (minutes/week)	Experimental group	91.16	188.75	116.77	192.13
	Control group	93.76	205.78	95.14	249.3
Moderate (minutes/week)	Experimental group	99.2	175.46	112.56	187.64
	Control group	95.78	155.42	104.76	169.77
Walking (minutes/week)	Experimental group	326.78	276.45	373.81	319.2
	Control group	354.2	287.94	366.19	258.31
Total (minutes/week)	Experimental group	517.14	640.66	603.14	698.97
	Control group	543.74	649.14	566.09	677.38

For Moderate PA: Control group Pre = 95.78, Post = 104.76, Experimental group Pre = 99.2, Post = 112.56 (Table 2). The odds ratio for Vigorous PA: is 1.152. The probability of Moderate PA after intervention in the experimental group was 15% higher than in the control group. This difference is also not statistically significant.

For Walking PA: Control group Pre = 354.2, Post = 366.19, Experimental group Pre = 326.78, Post = 373.81 (Table 2). The odds ratio for Vigorous PA: is 1.097. The probability of Walking PA after intervention in the experimental group was 10% higher than in the control group. However, this difference is also not statistically significant.

For Total PA: Control group Pre = 543.74, Post = 566.09, Experimental group Pre = 517.14, Post = 603.14 (Table 2). The odds ratio for Vigorous PA: is 1.203. The probability of Total PA after intervention in the experimental group was 20% higher than in the control group. However, this result does not reach statistical significance.

Overall, all these results are not statistically significant enough to determine the effectiveness of the intervention in relation to the level of physical activity.

### The effectiveness of the intervention regarding motivation

The obtained correlation results between the control and experimental groups for the magnitude of motivation (Table 3) indicate differences between the groups for different types of motivation. All four types of motivation are statistically significant, as the t values exceed the critical t value t_crit = 1.972 with df = 179 і α = 0.05.

**Table 3:** Correlation coefficients for motivation and breslow-day test results

Group	Motivation Type	Correlation	Squared Residuals	Correlation between Squared Residuals and Motivation	t
Experimental	Intrinsic	0.82	0.7056	-0.33	5.77*
Control		0.05	0.0121		
Experimental	Identified	0.79	0.2025	-0.16	4.90*
Control		0.41	0.0169		
Experimental	External	0.89	0.0484	-0.57	3.10*
Control		0.88	0.0196		
Experimental	Amotivation	0.32	0.0289	-0.19	-2.52*
Control		0.53	0.0049		

* Statistically significant at *p* < 0.05

Intrinsic Motivation: for the Experimental Group, the correlation is rather high (0.82), indicating a strong relationship between pre-and post-intervention levels of intrinsic motivation; for the Control Group, the correlation is low (0.05), suggesting a lack of a strong relationship between pre-and post-intervention levels of intrinsic motivation. Heterogeneity: -0.33 (high).

Identified Regulation: for the Experimental Group, the correlation is high (0.79), confirming a strong relationship between pre-and post-intervention levels of identified regulation; for the Control Group, the correlation is moderate (0.41), indicating a smaller, but existing, relationship between pre-and post-intervention levels of identified regulation in the control group. Heterogeneity: -0.16 (low).

External Regulation: for both the Experimental Group and the Control Group, a high correlation was observed (0.89 and 0.88 respectively) indicating a strong relationship between pre-and post-intervention levels of External Regulation. Heterogeneity: -0.57 (high).

Amotivation: for the Experimental Group, the correlation is low (0.32), indicating a weak relationship between pre-and post-intervention levels of amotivation; for the Control Group the correlation is moderate (0.53), suggesting a moderate relationship in the control group. Heterogeneity: -0.19 (low).

Thus, the obtained results indicate that the implementation of online training positively influences intrinsic and identified motivation, as well as external regulation, but may be less effective in reducing amotivation compared to traditional gym-based training. The heterogeneity of correlations suggests that the relationship between regression residuals and motivation may vary among different types of motivation and groups.

### Interrelation between participants’ demographic data, their physical activity, and jumping and running test results in the experimental group

**Table 4:** The correlation matrix

	Sex	Height (m)	Weight (kg)	Vigorous PA	Moderate PA	Walking	SJ	Sprint	PACER
Sex	-	0.227*	0.261					0.189	0.177
Height (m)	0.229*	-	0.053	-0.158	-0.095	-0.164	0.105	-0.111	-0.019
Weight (kg)	0.154	0.151	-	-0.224*	-0.005	-0.007	0.114	-0.094	-0.029
Vigorous PA (minutes / week)	-0.124	-0.122	-0.269	-	0.245	0.141	0.179	0.185	0.275*
Moderate PA (minutes / week)	-0.023	0.183	-0.167	0.177	-	0.196	0.041	0.061	0.176
Walking PA (minutes / week)	0.261*	-0.002	-0.133	0.128	0.659	-	0.036	0.087	0.133
SJ (w/kg)	0.125	0.117	0.102	0.114	0.156	0.117	-	0.076	0.112
Sprint 30 m (s)	0.156	-0.056	-0.107	0.258*	0.217*	0.181	0.552	-	0.107
PACER test (km/h)	0.189	-0.024	-0.025	0.247*	0.224*	0.184	0.113	0.134	-

* Statistically significant at *p* < 0.05

The upper triangular portion of the correlation matrix shows the results of pre-test comparisons, and the lower one shows the results of post-test comparisons (Table 4). The strongest relationship for both pre-and post-test is observed between Vigorous PA, Sprint, and PACER. We can also see a noticeable direct relationship between the variables Sex and Height and an inverse relationship between Weight and Vigorous PA.

## Discussion

The results of this study supported the previous findings of various researchers [25, 27, 31, 37, 38] regarding the effectiveness of technology-based PE interventions. Thus, researchers Mokmin and Jamiat [27] reported an increase in students’ PA and exercise motivation upon using the TRAINIME virtual fitness trainer app. They also claim that the majority of participants did not have a high level of PA before using the application. The students who participated in the study by Mokmin and Jamiat [27] highly enjoyed following the movements of virtual trainers and were able to successfully replicate them. The results of Mokmin and Jamiat [27] are based on The Cognitive Theory of Multimedia Learning (CTML). According to this theory, a properly constructed training scheme can interest a large number of students and, most importantly, help them achieve the best possible results. Mokmin and Jamiat [27] see their contribution in proving the effectiveness of using virtual trainers when training with a real coach is not possible. The increase in PA upon the intervention contradicts the results obtained in the current study. This can be explained, firstly, by the type of technologies used and, secondly, by the professional orientation of our sample. Hence, non-sports students are likely to have different PA results than those documented herein.

Kennedy et al. [38] analyzed the effectiveness of the Resistance Training for Teens intervention that lasted for 6 months. This intervention included: an interactive student seminar; a structured PA program focused on Resistance Training; lunchtime fitness sessions; and the use of mobile apps. The results obtained by Kennedy et al. [38] suggest that such intervention can improve the strength of upper body muscles and provide flexibility in autonomous PA motivation.

Similarly, Lee and Gao [31] evaluated how using mobile technology as part of PE classes impacts the PA and psychosocial beliefs of schoolchildren. Consequently, the hypothesis regarding an increase in PA as a result of using technology was not confirmed, which correlates with the findings of the current study. In addition, our study did not record any changes in PA over the course of the intervention, thus expanding the results documented by Lee and Gao [31]. In the same way, Lee and Gao [31] did not detect changes in the psychosocial beliefs of the intervention participants. The authors attribute this to the type of technology used during the intervention. However, we believe that this may be due to the short 2-week training period. The respondents of our study underwent online training for 12 weeks, which resulted in improved Intrinsic motivation and Identified regulation.

Another study by López-Sánchez et al. [33] analyzed the differences between body composition, physical fitness, and lifestyle behaviors in a sample of students from northern and southern Europe. The number of minutes per week spent in Vigorous PA was found to positively affect the largest number of considered variables [33]. Among these variables were high jump performance and PACER Final Speed. Although the sample consisted only of male students, the results of our study concern both men and women.

Marián et al. [39] described the experience of an 8-week jump squat training that involved moderately trained men (∼ 21 years old, ∼ 180 cm, ∼ 75 kg). They used jump squats with loads that allow repetitions to be performed ≥ 90% of maximum average power output [39]. An improvement in isometric half squat maximal force production (Fmax) and the positive association between Fmax and SJ (Squat Jumping) improvements were established. However, there were negative associations between Fmax and 50 m sprint time [39]. As a result, the implemented training was found effective for SJ, but not for sprinting. The authors explain this by the fact that the increase in maximum strength improves only certain elements of explosive sports performance. The participants of our study practiced both SJ and sprint skills, which is why their results differ from those obtained by Marián et al. [39].

With the help of Rosenborg FC elite soccer players, a study by Wisløff et al. [40] found a strong correlation between Fmax, sprinting, and jumping. All soccer players performed half squats as part of their regular strength training program. Additionally, 9 players performed five repetitions twice a week and increased weight by 5 kg each time they completed the weekly load. These players had a significantly higher maximal strength/power performance relation. Strength, power, and endurance are the most crucial characteristics for top soccer players, whereas power is heavily dependent on maximal strength. In contrast, the students who participated in our study were athletically trained and did not require high strength to maintain balance and control the ball as soccer players do. Therefore, we did not focus on that aspect during the intervention.

### Study limitations and future research prospects

The study involved a sample of male and female Chinese students who were in their second year of university studies. It also included a large number of variables and reliable tools for recording sports results. However, due to the lack of technical capabilities, other age groups and nationalities of students were not investigated. The small sample size also constitutes a certain limitation.

Thus, the obtained results cannot be fully applied to them. It should be noted that sports students attend theoretical and practical classes where they gain knowledge and perform physical activity. Moreover, the sports curriculum itself promotes a healthy and active lifestyle [41]. The adherence to physical activity by these students will be higher compared to students from other majors.

Future research should analyze the differences in PA, sports achievements, and motivation between students from different countries who use modern technology and train online. In addition, it is important to examine the age differences between students who prefer training in the gym and those who prefer home workouts.

## Conclusions

To sum up, no significant intervention effect on participants’ physical activity (PA) was observed in the study (RQ1). However, online training proved to be effective in terms of Intrinsic Motivation, Identified Regulation, and External Regulation but was less effective in reducing Amotivation compared to traditional gym-based training (RQ2). A strong direct correlation was found between the variables Vigorous PA, Sprint, and Progressive Aerobic Cardiovascular Endurance Run. Likewise, there was a significant correlation between the variables Sex and Height, and an inverse correlation between the variables Weight and Vigorous PA (RQ3). We would also like to note that the intervention (online training) was conducted to reinforce and not completely replace traditional gym training. Therefore, the key conclusion that can be drawn from this study is that the implemented online training can enhance gym training, as it has improved both academic performance and motivation of students. The implementation of additional professional online courses based on the example of “Sprinting Smarter, Sprinting Faster” is a great way to improve the PE efficiency of modern university students.

At the same time, the implemented course was found ineffective in increasing the students’ PA. Hence, it is necessary to search for more suitable means and methods that emphasize students’ well-being and personality-oriented motor activity. PE teachers, psychologists, and software developers should join together and find optimal solutions that would motivate students and help them achieve high sports results. The simplest solution can be to develop a mobile application aimed at implementing the goal-setting concept among professional athletes. Future research can expand the list of psychometric tests used for improving sports performance and PA when incorporating technological innovations.

## Data Availability

All data generated or analysed during this study are included in this published article.

## References

[1] Prensky MH. sapiens digital: From digital immigrants and digital natives to digital wisdom. Inn: J Onl Educ 2009;5(3):1–9. https://www.learntechlib.org/p/104264/

[2] Yu H, Kulinna PH, Lorenz KA (2018). An integration of mobile applications into physical education programs. Strategies.

[3] López-Valenciano A, Suárez-Iglesias D, Sanchez-Lastra MA, Ayán C (2021). Impact of COVID-19 pandemic on university students’ physical activity levels: an early systematic review. Front Psych.

[4] Wilson OW, Holland KE, Elliott LD, Duffey M, Bopp M (2021). The impact of the COVID-19 pandemic on US college students’ physical activity and mental health. J Phys Activ Heal.

[5] Goad T, Towner B, Jones E, Bulger S (2019). Instructional tools for online physical education: using mobile technologies to enhance learning. J Phys Educ Recr Dan.

[6] Kljajević V, Stanković M, Đorđević D, Trkulja-Petković D, Jovanović R, Plazibat K, Oršolić M, Čurić M, Sporiš G (2021). Physical activity and physical fitness among university students—A systematic review. Intern J Envir Res Publ Heal.

[7] Kumar A (2022). Gamification in training with next generation AI-virtual reality, animation design and immersive technology. J Exper Theor Artif Intell.

[8] Lounassalo I, Salin K, Kankaanpää A, Hirvensalo M, Palomäki S, Tolvanen A, Yang X, Tammelin TH (2019). Distinct trajectories of physical activity and related factors during the life course in the general population: a systematic review. BMC Publ Heal.

[9] Mitrović BJ, Janković R, Dopsaj M, Vučković G, Milojević S, Pantelić S, Nurkić M. How an eight-month period without specialized physical education classes affects the morphological characteristics and motor abilities of students of the Academy of Criminalistic and Police Studies. Fac Univ Ser: Phys Educ Spor. 2016;14(2):167–178. http://casopisi.junis.ni.ac.rs/index.php/FUPhysEdSport/article/view/1859

[10] Sáez I, Solabarrieta J, Rubio I (2021). Reasons for sports-based physical activity dropouts in university students. Int J Environ Res Public Health.

[11] Gil-Espinosa FJ, Nielsen-Rodríguez A, Romance R, Burgueño R (2022). Smartphone applications for physical activity promotion from physical education. Educ Inf Techn.

[12] Priyambada G, Prayoga AS, Utomo AWB, Saputro DP, Hartono R (2022). Sports app: digitalization of sports basic movement. Intern J Hum Mov Spor Scie.

[13] Schipperijn J, Cerin E, Adams MA, Reis R, Smith G, Cain K, Christiansen LB, van Dyck D, Gidlow C, Frank LD, Mitáš J, Pratt M, Salvo D, Schofield G, Sallis JF (2017). Access to parks and physical activity: an eight country comparison. Urb Urb Green.

[14] Rodrigues F, Monteiro D, Teixeira D, Cid L (2022). Understanding motivational climates in physical education classes: how students perceive learning and performance-oriented climates by teachers and peers. Curr Psych.

[15] Bortoli L, Bertollo M, Filho E, di Fronso S, Robazza C (2017). Implementing the TARGET model in physical education: effects on perceived psychobiosocial and motivational states in girls. Front Psychol.

[16] Cid L, Pires A, Borrego C, Duarte-Mendes P, Teixeira DS, Moutão JM, Monteiro D (2019). Motivational determinants of physical education grades and the intention to practice sport in the future. PLoS ONE.

[17] Huéscar Hernández E, Moreno-Murcia JA, Cid L, Monteiro D, Rodrigues F (2020). Passion or perseverance? The effect of perceived autonomy support and grit on academic performance in college students. Intern J Environ Res Publ Heal.

[18] Vasconcellos D, Parker PD, Hilland T, Cinelli R, Owen KB, Kapsal N, Lee J, Antczak D, Ntoumanis N, Ryan RM, Lonsdale C (2022). Self-determination theory applied to physical education: a systematic review and meta-analysis. J Educ Psych.

[19] Ryan RM, Deci EL, Maggino F (2022). Self-determination theory. Encyclopedia of quality of life and well-being research.

[20] Alamri H, Lowell V, Watson W, Watson SL (2020). Using personalized learning as an instructional approach to motivate learners in online higher education: Learner self-determination and intrinsic motivation. J Res Technol Educ.

[21] Ferrer J, Ringer A, Saville K, Parris MA, Kashi K (2022). Students’ motivation and engagement in higher education: the importance of attitude to online learning. High Educ.

[22] Fandiño FGE, Velandia AJS (2020). How an online tutor motivates E-learning English. Heliyon.

[23] Franck C, Grandi SM, Eisenberg MJ (2013). Taxing junk food to counter obesity. Am J Publ Heal.

[24] Gu X, Chen YL, Jackson AW, Zhang T (2018). Impact of a pedometer-based goal-setting intervention on children’s motivation, motor competence, and physical activity in physical education. Phys Educ Spor Pedag.

[25] Lu Y, Yu K, Gan X (2022). Effects of a SMART goal setting and 12-week core strength training intervention on physical fitness and exercise attitudes in adolescents: a randomized controlled trial. Intern J Envir Res Publ Heal.

[26] Mayer RE. Multimedia learning and games. In: Tobias S, Fletcher JD, editors. Computer games and instruction. Charlotte: IAP Information Age Publishing; 2011. pp. 281–305. https://psycnet.apa.org/record/2011-11269-010

[27] Mokmin NAM, Jamiat N (2021). The effectiveness of a virtual fitness trainer app in motivating and engaging students for fitness activity by applying motor learning theory. Educ Inf Techn.

[28] Fernández-Espínola C, Almagro BJ, Tamayo-Fajardo JA, Paramio-Pérez G, Saénz-López P (2022). Effects of interventions based on achievement goals and self-determination theories on the intention to be physically active of physical education students: a systematic review and meta-analysis. Sustainability.

[29] Sallis JF, Conway TL, Cain KL, Carlson JA, Frank LD, Kerr J, Glanz K, Chapman JE, Saelens BE (2018). Neighborhood built environment and socioeconomic status in relation to physical activity, sedentary behavior, and weight status of adolescents. Prev Med.

[30] Behzadnia B, Mohammadzadeh H, Ahmadi M (2019). Autonomy-supportive behaviors promote autonomous motivation, knowledge structures, motor skills learning and performance in physical education. Curr Psych.

[31] Lee JE, Gao Z (2020). Effects of the iPad and mobile application-integrated physical education on children’s physical activity and psychosocial beliefs. Phys Educ Sport Pedag.

[32] Athlete Factory. YAP Training Videos Seated Box Jump with Weight 12LB Medicine Ball. https://www.youtube.com/watch?v=MjjpKFSqcMc

[33] López-Sánchez GF, Radzimiński Ł, Skalska M, Jastrzębska J, Smith L, Wakuluk D, Jastrzębski Z (2019). Body composition, physical fitness, physical activity and nutrition in Polish and Spanish male students of sports sciences: differences and correlations. Intern J Envir Res Publ Heal.

[34] Kotzamanidis C (2006). Effect of plyometric training on running performance and vertical jumping in prepubertal boys. J Stren Condit Res.

[35] Dinger MK, Behrens TK, Han JL (2006). Validity and reliability of the International Physical Activity Questionnaire in college students. Am J Heal Educ.

[36] Guay F, Vallerand RJ, Blanchard C (2000). On the assessment of situational intrinsic and extrinsic motivation: the situational motivation scale (SIMS). Motiv Emot.

[37] Grima JS, Blay MG (2016). Perfil cardiovascular en estudiantes de ciencias de la Actividad Física Y Del Deporte, estudiantes de otras disciplinas y trabajadores en activo. Medic Gen Fam.

[38] Kennedy SG, Smith JJ, Morgan PJ, Peralta LR, Hilland TA, Eather N, Lonsdale C, Okely AD, Plotnikoff RC, Salmon J, Dewar DL, Estabrooks PA, Pollock E, Finn TL, Lubans DR (2018). Implementing resistance training in secondary schools: a cluster randomized controlled trial. Med Scie Spor Exerc.

[39] Marián V, Katarína L, Dávid O, Matúš K, Simon W. Improved maximum strength, vertical jump and sprint performance after 8 weeks of jump squat training with individualized loads. J Spor Scie Medic. 2016;15(3):492–500. https://www.ncbi.nlm.nih.gov/pmc/articles/PMC4974862/PMC4974862 27803628

[40] Wisløff U, Castagna C, Helgerud J, Jones R, Hoff J (2004). Strong correlation of maximal squat strength with sprint performance and vertical jump height in elite soccer players. Br J Spor Med.

[41] Cohen J (1988). Statistical power analysis for the behavioral sciences.

